# Comparison of plasma lipoprotein profiles and malondialdehyde between hyperlipidemia dogs with/without treatment

**DOI:** 10.1186/1746-6148-10-67

**Published:** 2014-03-14

**Authors:** Gebin Li, Koh Kawasumi, Yuki Okada, Shingo Ishikawa, Ichiro Yamamoto, Toshiro Arai, Nobuko Mori

**Affiliations:** 1 Department of Veterinary Bioscience, School of Veterinary Medicine, Nippon Veterinary and Life Science University, 1-7-1 Kyonancho, Musashino, Tokyo 180-8602, Japan

**Keywords:** Dog, Hyperlipidemia, Lipid profiles, Malondialdehyde, Prevention, Screening criteria

## Abstract

**Background:**

The aim of this study is to compare metabolic parameters, malondialdehyde as a lipid oxidation marker, and lipid profiles between dogs with untreated hyperlipidemia and hyperlipidemia with treatment, in order to examine the usefulness of malondialdehyde and lipid profiles as diagnostic parameters at early stages of hyperlipidemia.

**Results:**

Dog samples were collected from four different veterinary clinics across Japan from March to June 2013. They were separated into three groups: control, untreated hyperlipidemia based on temporally screening, and hyperlipidemia with current anti-hyperlipidemic (statins and fibrates) treatment. Triglyceride levels of untreated hyperlipidemia dogs were significantly higher than those of control dogs. ALT levels of hyperlipidemic dogs with treatment were the highest among three groups. VLDL and LDL of both cholesterol and triglyceride of untreated hyperlipidemia dogs were the highest among three groups. HDL_1_ levels in triglyceride of hyperlipidemia dogs with treatment were significantly higher than those of control and untreated hyperlipidemia dog. Malondialdehyde concentrations of untreated hyperlipidemia dogs were significantly higher than those of control and hyperlipidemic dogs with treatment.

**Conclusions:**

In this study, dogs with untreated hyperlipidemia clearly showed abnormal lipid status, whereas hyperlipidemic dogs under anti-hyperlipidemia treatment showed more normal lipid status suggesting the effectiveness of the therapy. Anti-hyperlipidemics (statins and fibrates) for dogs are also effective in relieving elevated levels of lipids and lipid oxidation. Plasma lipid (triglyceride and cholesterol) profiles and malondialdehyde are useful diagnostic tools for identifying early stages of untreatment hyperlipidemia in dogs.

## Background

Aberrations in plasma cholesterol and triglyceride levels are indicative of diseases associated with obesity and diabetes mellitus [1]. Mounting evidences have suggested the increasing recognition of clinical importance of hyperlipidemia in dogs. Hyperlipidemia causes other disorders such as pancreatitis, hypotiroidism [2,3]. However, many of hyperlipidemia dogs physically appear healthy and do not usually exhibit any symptoms. Therefore, many veterinary practitioners and dog owners exhibit little interest in unsymptomatic lipid metabolism abnormality. Only when the disease progresses and the dog become severely hyperlipidemic, they initiate therapy with anti-hyperlipidemic drugs. In 2012, we set the new screening criteria for detecting early stages of hyperlipidemia in dogs [4]. When we evaluated apparently healthy dogs using these criteria, 23.7% dogs (9 of healthy 38 dogs) were diagnosed with hyperlipidemia. We think that the use of hyperlipidemia screening is valuable in early diagnosis, which in turn, allows for an implementation of early interventions such as diet change, exercise, and more frequent veterinary check-ups. In humans, plasma lipid profile analysis can be useful in diagnosing lipid metabolic disorders. Lipoproteins are believed to play important roles in energy and lipid metabolism of animals, and can reflect metabolic changes. Alterations in the dog’s lipoprotein cholesterol fractions are possibility of aberrations of lipid metabolism due to hyperlipidemia and/or obesity as in human [5].

Hyperlipidemia also induces oxidative stress, and malondialdehyde (MDA) is one of the end products in lipid peroxidation. Plasma MDA levels increased markedly in animals with obesity and diabetes mellitus and increased MDA indicated elevations of lipid oxidation in tissues [6]. Humans with apparent increase in Malondialdehyde-Modified LDL were shown to be more predisposed to developing arterosclerosis. However it should be noted, dog’s lipoprotein density profiles and lipid metabolism are different from those of humans [5,7].

The aim of this study is twofold. First is to reveal how the effect of the anti-hyper lipidemic therapy on lipid profiles of hyperlipidemia dogs. There are 2 types of hyperlipedemia dogs; Fisrt hyperlipidemia dog group is classified as untreated hyperlipedemia, and another group of hyperlipidemia dogs managed with lipid-lowering drugs was classified as hyperlipidemia with current treatment.

Second is to compare metabolic parameters, MDA as a lipid oxidation marker, and lipid profiles between dogs with untreated hyperlipidemia, and hyperlipidemia with treatment in order to examine the usefulness of MDA and lipid profiles as diagnostic parameters at early stages of hyperlipidemia.

## Methods

### Animals

21 dog samples were collected from four different veterinary clinics across Japan (Ibaraki, Kanagawa) from March to June 2013. The test subjects included 4 Miniature Dachshunds, 3 Toy Poodles, 2 Golden Retrievers, and 2 Shih Tzus and so on. Breeds generally known to exhibit hyperlipidemia such as Miniature Schunauzers and Shetland Sheepdogs [8,9] were excluded from our subjects. 13 dogs (age: median 8.0 years old; number of females (F): 8; number of males (M): 5; body weight: median 5.6 kg) were classified as healthy control subjects by each clinical veterinarian. 4 dogs (age: median 12.5 years; M: 4; body weight: median 8.9 kg) under anti-hyperlipidemic therapy (statins and fibrates) were diagnosed as severely hyperlipidemic. The remaining 4 dogs (age: median 9.5 years; F: 3; M: 1; body weight: median 7.7 kg) were diagnosed as hypelipidemic based on the screening of hyperlipidemia as follow [4]. A subject may be classified as hyperlipedemic if the biochemical analysis of the plasma shows any two of the following 3 factors:

1) Elevated triglyceride level >165 mg/dL

2) Elevated total cholesterol level >200 mg/dL

3) Elevated free fatty acids level >1.5 mEq/L

All dog owners gave us written informed consent before enrolment in this study. This study was approved by the Nippon Veterinary and Life Science University Animal Research Committee.

### Blood sampling and plasma metabolite analysis

Postprandial blood samples were collected from the forelimb vein into heparinized plastic tubes, and plasma was recovered via centrifugation at 4°C and stored at -25°C until further use. Plasma glucose, triglycerides (TG), total cholesterol (T-cho), total protein, aspartate aminotransferase, and alanine aminotransferase (ALT), alkaline phosphatase (ALP), albumin, albumin verses globulin ratio, total bilirubin, total bile acids, γ-GTP were measured by auto-analyzer (Monolis, Inc, Tokyo, Japan) using the manufacturer’s reagents. NEFA, adiponectin, insulin and MDA were determined with NEFA-C test Wako (Wako Pure Chemical Industries, Inc., Tokyo), Dog adiponectin ELISA kit (Circulex Co., Ltd, Nagano), and Lbis dog Insulin kit (Shibayagi Co., Gunma), Malondialdehyde Assay kit, (Northwest Life Science Specialties LLC, Vancouver, WA) respectively.

In order to examine lipoprotein fractions of cholesterol and triglyceride for all dogs, we performed biphasic agarose gel electrophoresis method using commercial Quickgel Lipo gels (Helena Laboratories, Saitama). Lipoproteins were separated by electrophoretic technique and analyzed using a Helena Laboratories Epalyzer 2 Electrophoresis processing Analyzer. Lipoprotein fractions were assessed and analyzed using Edbank III analysis software (Helena Laboratories, Saitama, Japan). Lipoprotein electrophoresis pattern consisted of 4 bands. The bands from left to right were: α1-migrating lipoproteins (HDL_2_ and HDL_3_), α2-migrating lipoproteins (HDL_1_), β-migrating lipoproteins (LDL and VLDL), and κ band (small% of chylomicrons) remaining at the origin (Mori et al., [5]).

### Statistical analysis

Values were expressed in medians with inter-quartile range (25th Percentile and 75th Percentile). Statistical significance was determined by One-way Ananysis of Variance by Ranks among groups. All tests were performed using Sigmaplot version.11.2 (Systat Software Inc., San Diego, CA, USA). The significance level was set at P < 0.05.

## Results

**Figure 1:**
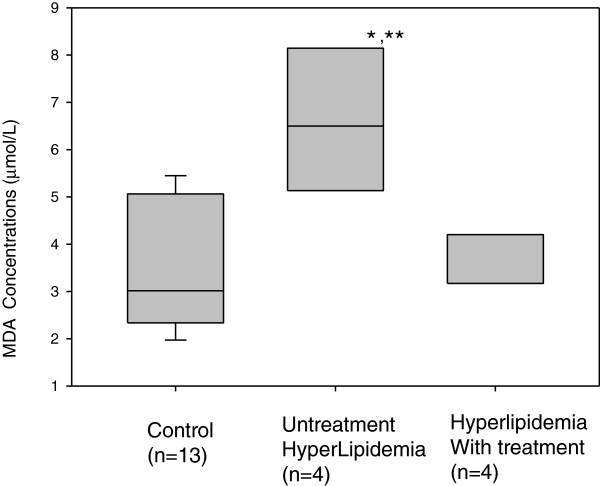
**Change of MDA concentrations among control, hyperlipidemia dogs with/without treatment.** Graph is expressed as median with inter-quartile range (25th Percentile and 75th Percentile). * Significantly different (p < 0.05) from the values of Control group by Kruskal-Wallis One Way Analysis of Variance on Ranks. ** Significantly different (p < 0.05) from the values of hyperlipidemia with treatment group by Kruskal-Wallis One Way Analysis of Variance on Ranks.

As shown in Table 1, triglycerides level of untreated hyperlipidemia group was significantly higher than those of control group. ALT level of hyperlipidemia with treatment group was the highest among three groups. ALP level of hyperlipidemia dog with treatment was significantly higher than those of control and untreated hyperlipidemia dogs. Although no significant difference in total cholesterol concentration was observed, total cholesterol level of hyperlipidemic dogs with treatment tended to be higher compared to the other groups. VLDL and LDL in cholesterol and triglyceride of untreated hyperlipidemia dogs were the highest among three groups. HDL_1_ level in triglyceride of hyperlipidemia with treatment group was significantly higher than those of control and untreated hyperlipidemic groups. Chylomicron level in triglyceride of untreated hyperlipidemia dogs was higher than those of control and hyperlipidemic dogs with treatment. As shown in Figure 1. MDA concentration s of untreated hyperlipidemia dogs was the highest among three groups.

**Table 1:** Comparison of plasma biomarker levels and lipoprotein profiles among control, hyperlipidemia dogs with/without treatment

**Group (n)**	**Control (13)**	**Hyperlipidemia**	
		**Untreatment (4)**	**With treatment (4)**
Age	8.0 (2.8-9.3)	9.5 (6.0-11.5)	12.5 (12.-15.0)
Body condition score	3.0 (3.0-4.0)	3.5 (2.3-4.8)	3.0 (3.0-4.0)
Body weight (kg)	5.6 (4.3-14.5)	7.7 (3.0-21.3)	8.9 (5.6-26.9)
Total choresterol (mg/dL)	240 (170–351)	241 (231–358)	367 (347–390)
Triglycerid (mg/dL)	48 (28–163)	252 (196–311)*	81 (61–88)
Total protein (g/dL)	6.8 (6.4-7.4)	7.5 (6.7-8.0)	7.3 (7.1-7.7)
Albmin (g/dL)	3.3 (3.0-3.4)	3.4 (3.0-3.8)	3.3 (3.0-3.6)
Albumin/globulin ratio	0.88 (0.79-0.97)	0.83 (0.80-0.89)	0.78 (0.76-0.88)
Total bilirubin (mg/dL)	0.1 (0.1-0.2)	0.2 (0.1-0.2)	0.1 (0.1-0.2)
AST (IU/L)	33 (24–39)	35 (20–38)	33 (26–45)
ALT (IU/L)	44 (37–67)	40 (24–65)**	123 (75–315)*
ALP (IU/L)	127 (72–218)	335 (101–2259)	877 (318–1113)*
γ-GTP (IU/L)	6.1 (3.9-7.7)	5.1 (2.8-7.7)	7.7 (6.0-35.8)
Total bile acid (mg/dL)	5.7 (2.5-23.5)	15.8 (6.4-104.3)	10.4 (6.2-31.2)
Glucose (mg/dL)	90 (81–110)	102 (96–109)	103 (90–375)
NEFA (mEq/L)	0.78 (0.64-0.97)	0.73 (0.55-1.14)	0.52 (0.41-0.65)
Insulin (ng/mL)	1.1 (0.8-1.7)	0.7 (0.6-1.1)	2.4 (1.2-2.5)
Adiponectin (μg/mL)	18.4 (15.9-30.1)	31.9 (17.1-38.9)	23.8 (19.0-25.9)
Cholesterol profiles			
HDL2,3 (mg/dl)	191.8 (143.1-225.0)	191.2 (167.2-240.0)	229.7 (215.8-242.8)
HDL1 (mg/dl)	31.1 (24.3-85.5)	41.7 (25.2-124.4)	119.9 (96.3-136.6)
VLDL/LDL (mg/dl)	5.8 (3.3-14.4)	22.5 (16.5-35.7)*	21.5 (7.7-35.9)
CM (mg/dl)	0.0 (0.0-0.2)	1.5 (0.0-6.7)	0.0 (0.0-0.0)
Triglyceride profiles			
HDL2,3 (mg/dl)	0.0 (0.0-0.0)	0.0 (0.0-0.0)	0.0 (0.0-0.2)
HDL1 (mg/dl)	0.5 (0.0-1.8)	0.9 (0.75-5.0)	3.0 (1.9-6.5)*
VLDL/LDL (mg/dl)	47.5 (28.1-108.4)	193.1 (119.0-264.5)*,**	67.9 (59.7-72.3)
CM (mg/dl)	0.5 (0.0-48.7)	58.6 (0.0-119.3)	5.6 (0.0-14.5)

Values are presented as median. Numbers in parentheses present from 25% to 75% value.

*Significantly different (p < 0.05) from the values of Control group by Kruskal-Wallis One Way Analysis of Variance on Ranks.

**Significantly different (p < 0.05) from the values of hyperlipidemia with treatment group by Kruskal-Wallis One Way Analysis of Variance on Ranks.

All dogs were also determined by palpation and inspection on with a body condition score (BCS: a 5 point scale) the five-point scale: (1) very thin, (2) underweight, (3) ideal, (4) overweight, and (5) obese.

## Discussion

Various diagnostic criteria for hyperlipidemia have already been established. The first states plasma total cholesterol level of over 300 mg/dl as a criterion [8], and another sets both plasma triglyceride and plasma cholesterol levels to be over 500 mg/dl [3]. The above methods only detect dogs which are in severe hyperlipidemic condition and in need of clinical interventions. Our screening methods aim to detect early stages of hyperlipidemia in dogs that may be missed by veterinarinary practitioners. In current study, abnormal lipid satus in plasma was clearly detected in untreated hyperlipidemia group. In this study, most of the hyperlipidemic dogs were under anti-hyperlipidemic therapy as recommended by their veterinarians. The therapy appeared to be fully effective since triglyceride level in hyperlipidemia with treatment group was lower than those of untreated hyperlipidemia group and there was no significant difference in plasma cholesterol levels among three groups. At the time of the blood draw, three of four hyperlipidemic dogs were on fibrates and one dog was on statins. The fibrates are considered the most effective hypertriglyceridemia drugs [10] and the statins are a class of LDL-cholesterol lowering drugs [11,12]. ALT levels in hyperlipidemic dogs with treatment were expected to be elevated due to hepatic metabolism of these drugs. HDL_1_ is a large floating particle which plays an important role in reverse cholesterol transport. LDL functions similarly as HDL 1, and is unique to dogs [5]. HDL_1_ fractions of both cholesterol and triglycerides in hyperlipidemia with treatment group were the highest among three groups. Additionally, we confirmed correlations between MDA and lipid profiles for each group. MDA level of normal group showed strongly positive correlations with triglycerides level, VLDL of triglycerides (TG r = 0.89, VLDL of TG r = 0.82). Remarkably, MDA level of untreated hyperlipidemia group showed strongly positive correlations with a lot of lipid profiles (T-Cho r = 0.83, HDL_2_ and HDL_3_ of T-cho r = 0.86, HDL_1_ of T-cho r = 0.72, VLDL and LDL of T-cho r = 0.97, HDL1 of TG r = 0.78, VLDL and LDL of TG r = 0.79). Adversely MDA of hyperlipidemia with treatment correlated negatively with total cholesterol level (T-cho, r = -0.75). The highest value of coefficient of correlation was seen between MDA and VLDL/LDL of total cholesterol.

Anti-hyperlipidemic drugs were also effective in reducing lipid oxidation. MDA concentration in untreated hyperlipidemic dogs was the highest among three groups. MDA is an end product of lipid peroxidation, and MDA-modified LDL (MDA-LDL) is generated via lipid peroxidation or during platelet aggregation. Both statins and fibrates therapies had a positive effect on reducing serum MDA-LDL concentrations in human lipid metabolic mechanism [13,14]. Also statins and fibrates in dogs are effective in relieving the increased lipids and lipid oxidation. Untreated hyperlipidemia group showed significantly higher plasma triglycerides concentration than control and hyperlipidemia with treatment groups. Associated VLDL and LDL in lipoprotein fractions were also higher than those of control and hyperlipidemia with treatment groups. As untreated hyperlipidemia dogs showed abnormality in lipid metabolism, our new criteria in hyperlipidemia is shown to be beneficial in metabolic disease prevention. Xenoulis et al. [15] also reported in current paper that lipoprotein profiles in dogs could potentially be useful as diagnostic tools in idenfication of dogs suspected of having lipoprotein abnormalities.

Unfortunately, in this study, there was an insufficient number of with/ without treatment hyperlipidemic dogs. The small number of dogs may result in low statistical power, hence results and conclusions need to be interpreted with care. However, this study showed that changes in lipid metabolism are reflected in liver. In the future, we hope to add more sample numbers and to repeat our study using different stages of hyperlipidemia dogs and establish base values of lipid profiles and MDA concentrations as new markers in hypelipidemia screening in order to validate our results.

## Conclusions

In this study, dogs with untreated hyperlipidemia clearly showed abnormal lipid status, whereas hyperlipidemic dogs under anti-hyperlipidemia treatment showed more normal lipid status suggesting the effectiveness of the therapy. Plasma lipid (triglycerides and cholesterol) profiles and MDA may be useful as diagnostic tools for early stages of hyperlipidemia dogs.

## Competing interest

The authors have no financial or personal relationships with other people or organizations related to this work.

## Authors’ contributions

GL drafted the manuscript and carried out the vast majority of the experimental work. KK and YO were involved in revising the manuscript, with data and statistical analysis. IY was involved with analysis of plasma metabolites from sera samples. TA participated in the study design and its coordination. NM conceived of the study, and participated in its design and coordination. All authors read and approved the final manuscript.
